# The effects of spin-orbit coupling on optical properties of monolayer $\text {MoS}_{2}$ due to mechanical strains

**DOI:** 10.1038/s41598-023-28258-z

**Published:** 2023-01-20

**Authors:** H. Rezania, M. Abdi, B. Astinchap, E. Nourian

**Affiliations:** 1 Department of Physics, Razi University, Kermanshah, Iran; 2 Department of Physics, Faculty of Science, University of Kurdistan, Sanandaj, Kurdistan 66177-15175 Iran; 3 Research Center for Nanotechnology, University of Kurdistan, Sanandaj, Kurdistan 66177-15175 Iran

**Keywords:** Electronic properties and materials, Physics

## Abstract

We have studied the optical conductivity of a quasi two-dimensional $\text {MoS}_{2}$ in the presence of external magnetic field and spin-orbit coupling. Specially, we address the frequency dependence of optical conductivity due to spin-orbit interaction. Using linear response theory the behavior of optical conductivity has been obtained within Green’s function method. We have also considered the effects of uniaxial and biaxial in-plane strain on the optical absorption of $\text {MoS}_{2}$ layer. In the absence of external magnetic field with negative uniaxial strain parameter, optical conductivity includes Drude weight at zero frequency limit while Drude weight vanishes for $\text {MoS}_{2}$ layer under positive uniaxial strain. Our results show that the increase of uniaxial positive strain parameter causes to move the position peak to the higher frequencies. In contrast to uniaxial strain case, the Drude weight in optical conductivity appears at positive biaxial strain value 0.15. Also we have studied the effects of magnetic field, electron doping, hole doping in the presence of spin-orbit coupling on frequency dependence of optical conductivity of $\text {MoS}_{2}$ in details. The magnetic field dependence of optical absorption shows a monotonic decreasing behavior for each value of temperature in the absence of strain parameter.

## Introduction

Graphene was synthesized as the first two-dimensional nanostructure in 2004 year^1,2^, due to its attractive physical properties^3,4^, which led to the attention of other 2D-nanostructures such as transition metal dichalcogenides (TMDs)^5,6^, Phosphorene^7,8^, Silicene^9^, Germanene^10^, and Stanene^11^, etc.^12,13^. One of the most important groups of 2D-nanostructures is TMDs, which include two groups of a transition metal atoms (Mo, W, etc.) and chalcogen atoms (S, Se, etc.). TMDs due to their very interesting and great promise properties have made it possible to use them in electronic and spintronic applications^14,15^. One of the most important members of this group is $\text {MoS}_{2}$. $\text {MoS}_{2}$ bulk has an indirect bandgap of 1.3 eV, and the monolayer is of a direct bandgap of 1.8 eV^16,17^. The $\text {MoS}_{2}$ monolayer has a honeycomb lattice structure in which each Mo atom is covalently sandwiched between two layers of S atoms. Recently, $\text {MoS}_{2}$ is more appropriate for use in transistor field effect^18–20^, photovoltaic^21^, spintronic^22^, and valleytronic devices^23,24^. A series of theoretical studies on the electronic band structure of $\text {MoS}_{2}$ have been done by using first-principle calculations^25,26^. Researchers have shown that applying strain to the monolayer $\text {MoS}_{2}$ changes its band-gap transition from direct to indirect^27–29^. In semiconductors such as $\text {MoS}_{2}$, excitons are formed because electron-hole pairs interact with each other by Coulomb attraction, that excitons determine the optical properties of $\text {MoS}_{2}$^30–32^. For instance, experimentations like photoluminescence^33^ and second harmonic generation^34,35^ are strongly impressed by excitons. Jia et al. studied the structural and optical properties of multilayer $\text {MoS}_{2}$ by using the first-principles method. They showed that the increased number of layers leads to small changes in the direct energy gap near point K (weak interlayer coupling) and larger changes near point $\Gamma$ (stronger interlayer coupling). Therefore caused a small redshift in the threshold energy and a noticeable redshift at the end of total joint density of states^36^. In recent years, the optical properties of $\text {MoS}_{2}$ have been studied by experimental^37,38^ and theoretical^39–41^ methods. The results show that $\text {MoS}_{2}$ has significant applications in the optoelectronics industry^42,43^.

The intrinsic spin-orbit coupling plays an important role on the topological and thermal properties of honeycomb structures such as $\text {MoS}_{2}$ plane. Such coupling arises from perpendicular electric field or interaction with a substrate. Based on extensive theoretical studies, opening a bulk gap in band structure of $\text {MoS}_{2}$ plane originates from both spin orbit coupling and exchange field factors so that leads to the quantum spin hall effect^44,45^. In the presence of spin-orbit coupling, a compressive biaxial in-plane strain and perpendicular tensile strain can lead even to a topological phase transition^46^. The strong spin-orbit coupling indeed leads to a different spin-polarization of the valence band. Thus several degrees of freedom are strongly entangled in TMDs^47,48^. Tuning the spin-orbit coupling of mechanical deformation has been explored in conventional GaAs based semiconductors and quantum wells where a linear strain dependence is found in the presence of this coupling^49,50^.

Strain effects have been studied to other 2D crystals and recently the possibility to tune the band gap with strain has been experimentally proven for $\text {MoS}_{2}$^51,52^. The piezoelectricity phenomenon have been investigated for monolayer $\text {MoS}_{2}$^53^ so that it seems to require a deeper understanding of the effect of external nonuniform strain on the physical properties of semiconductor TMDs.

The purpose of this paper is to provide a Slater Koster tight binding model^26,48^ including intrinsic spin-orbit interaction for studying the optical properties of $\text {MoS}_{2}$ monolayer in the presence of magnetic field perpendicular to the plane. Firstly, we introduce the Hamiltonian of the monolayer $\text {MoS}_{2}$ by tight-binding and then the Green’s function has been obtained using the calculated energy levels. We will examine the results of optical absorption under electron/hole doping, external magnetic field, strain, and temperature. The effects of homogenous strains on the optical properties of $\text {MoS}_{2}$ plane are investigated using linear response theory. In other words, the effects of these strains on frequency dependence of optical absorption of $\text {MoS}_{2}$ layer are investigated in details. Using the suitable hopping integral and strain parameters, the band dispersion of electrons on the structure has been calculated. For calculating the optical absorption we have exploited the linear response theory in the context of Kubo formula. Green’s function approach has been exploited to obtain the optical absorption of $\text {MoS}_{2}$ monolayer. The effects of external magnetic field, biaxial strains and spin-orbit coupling strength on the frequency behavior of optical absorption of $\text {MoS}_{2}$ monolayer have been studied. Also we discuss and analyze to show how spin orbit coupling, longitudinal magnetic field and biaxial strain values affect the photon frequency behavior of optical absorption of $\text {MoS}_{2}$ plane. Finally the dependence of optical absorption on biaxial strain values and spin-orbit coupling is addressed.

The parts remained of this article is planned as follows. In “Model Hamiltonian and formalism” section, we determine the tight-binding model and the Green function for the $\text {MoS}_{2}$ monolayer in the presence of the Zeeman effect, spin-orbital coupling and biaxial strain. We express our calculation of the optical absorption in Section ”[Sec Sec3]”. Numerical results of the optical absorption under the influence of various factors are present in Discussion and conclusion. In Section "Discussion and conclusion", includes a summary of the results and discussions.

## Model Hamiltonian and formalism

**Figure 1:**
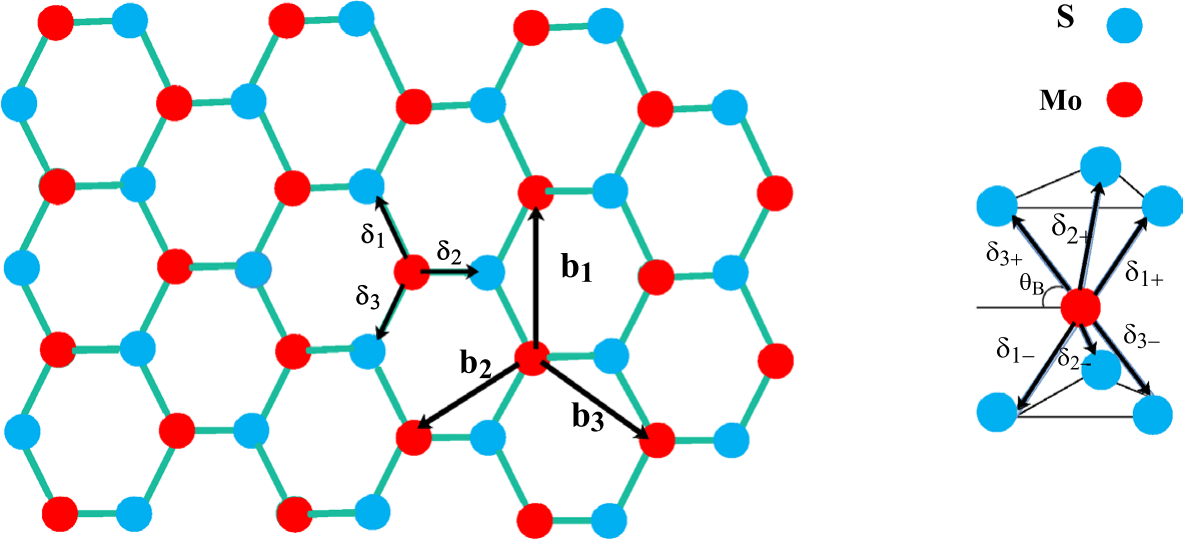
Left panel: A top view schematic of single layer $\text {MoS}_{2}$ lattice structure. Blue(red) circles indicate Mo(S) atoms. $\textbf{b}_{i=1,2,3}$ show the vectors connecting next nearest neighbor lattice sites while $\delta _{i=1,2,3}$ show vectors connecting nearest neighbor lattice sites. Right panel: Side view of the lattice structure are seen where Mo atom is surrounded by six S atoms.

$\text {MoS}_{2}$ monolayer consists of one layer of Mo atoms surrounded by two layers of S atoms in such a way that each Mo atom is coordinated by six S atoms in a trigonal prismatic geometry and each S atom is coordinated by three Mo atoms. The side and top views of the lattice structure where Mo atom is surrounded by six S atoms unit cell of $\text {MoS}_{2}$ has been indicated in Fig. 1a also we have sketched a top view of the crystal structure of $\text {MoS}_{2}$ in Fig. 1b.

In order to investigate the effect of homogenous strain and spin-orbit coupling on the electronic band structure and optical properties of monolayer $\text {MoS}_{2}$, we apply a Slater Koster tight binding model^54^ for describing the electron dynamics in the structure. This tight binding model Hamiltonian contains the relevant orbital character in the valence and conduction band. Also this model apllied for the single layer $\text {MoS}_{2}$ uses an orbital basis which includes $d_{3z^{2}-r^{2}}, d_{xy}$ and $d_{x^{2}-y^{2}}$ orbitals of the Mo, and $p_{x}$ , $p_{y}$ and $p_{z}$ orbitals of the atom S. In order to clarify the symmetric and antisymmetric hybridization of p-orbitals of S atoms localized on up and down layers, we use the definitions $p_{x}^{S}=1/\sqrt{2}(p^{u}_{x}+p^{b}_{x})$, $p_{y}^{S}=1/\sqrt{2}(p^{u}_{y}+p^{b}_{y})$ and $p_{z}^{A}=1/\sqrt{2}(p^{u}_{z}-p^{b}_{z})$. The relevant physics of monolayer $\text {MoS}_{2}$ around the gap is covered by a smaller subspace, which can be obtained by performing an appropriate unitary transformation that transform the *P* orbitals of the top and bottom S layers into their symmetric and antisymmetric combinations with respect to the *z* axis. For the single-layer case, the resulting 11-band model can be decoupled in six bands with even symmetry under inversion transformation *z* to $-z$, and five bands with odd symmetry^26,55,56^. Therefore, the conduction band minimum is mainly formed from orbitals $d_{3z^{2}-r^{2}}$ of Mo atoms and the valence band maximum is constructed from orbitals $d_{x^{2}-y^{2}}$ and $d_{xy}$ of Mo atoms with mixing of orbitals $p_{x},p_{y}$ from S atoms^57,58^ in both cases. Therefore we can consider the following basis orbitals for making Hilbert space as

1 $$\begin{aligned} \{|d_{3z^{2}-r^{2}}\rangle ,|d_{x^{2}-y^{2}}\rangle , |d_{xy}\rangle ,|p_{x}^{Sym}\rangle ,|p_{y}^{Sym}\rangle ,|p_{z}^{Anti}\rangle \}. \end{aligned}$$

The tight-binding model for $\text {MoS}_{2}$ monolayer in second quantizaton representation is given by

2 $$\begin{aligned} H^{TB}= & {} \sum _{i,\alpha }\epsilon ^{S}_{i,\alpha }a^{\dag }_{i,\alpha }a_{i,\alpha }+\sum _{i,\beta }\epsilon ^{Mo}_{i,\beta }b^{\dag }_{i,\beta }b_{i,\beta } +\sum _{\langle i,j\rangle ,\alpha ,\beta }t^{Mo-S}_{ij,\alpha \beta }a^{\dag }_{i,\alpha }b_{j,\beta } +\sum _{\langle \langle i,j\rangle \rangle ,\beta ,\beta '}t^{Mo-Mo}_{ij,\beta \beta '}b^{\dag }_{i,\beta }b_{j,\beta '}\nonumber \\+ & {} \sum _{\langle \langle i,j\rangle \rangle ,\alpha ,\alpha '}t^{S-S}_{ij,\alpha \alpha '}a^{\dag }_{i,\alpha }a_{j,\alpha '}+h.c., \end{aligned}$$

where $a^{\dag }_{i,\alpha }$ creates an electron in the atomic orbital $\alpha =p_{x}^{Sym},p_{y}^{Sym},p_{z}^{Anti}$ of S atom located in the *i*th unit cell. Also $b^{\dag }_{i,\beta }$ implies the creation operator of electron in the atomic orbital $\beta =d_{3z^{2}-r^{2}},d_{x^{2}-y^{2}},d_{xy}$ of Mo atom located in the *i*th unit cell. $t^{Mo-Mo}_{ij,\beta \beta '}$ introduces the hopping amplitude of electron between two Mo atoms that are located in next nearest neighbor lattice sites with atomic orbitals $\beta ,\beta '$ . $t^{S-S}_{ij,\alpha \alpha '}$ introduces the hopping amplitude of electron between two S atoms that are located in next nearest neighbor lattice sites with atomic orbitals $\alpha ,\alpha '$. $t^{Mo-S}_{ij,\alpha \beta }$ denotes hopping amplitude of electron between Mo and S atoms that are located in nearest neighbor lattice sites with atomic orbitals $\alpha ,\beta$. Also we have introduced $\langle i,j\rangle$ and $\langle \langle i,j\rangle \rangle$ for nearest neighbor and next nearest neighbor lattice sites respectively. $\epsilon ^{S}_{i,\alpha }$ refers to on-site energy of electron with atomic orbital $\alpha$ of S atom located in *i* th lattice site. In other hand, on-site energy of electron with atomic orbital $\beta$ of Mo atom located in *i* th lattice site is presented by $\epsilon ^{Mo}_{i,\beta }$.

The parameters $\epsilon ^{Mo}_{i,\beta }$ in Eq. 2) with different atomic orbital basis, i.e. $\beta =d_{3z^{2}-r^{2}},d_{x^{2}-y^{2}},d_{xy}$ are the matrix elements of a diagonal 3$\times$3 matrix, $\epsilon ^{Mo}_{i}$. This diagonal matrix is given by

3 $$\begin{aligned} \epsilon ^{Mo}_{i}= & {} \left( \begin{array}{ccc} \Phi _{0}&{} 0&{}0 \\ 0&{} \Phi _{1}&{}0 \\ 0&{} 0&{}\Phi _{2}\\ \end{array} \right) ,\nonumber \\ \Phi _{0}\equiv & {} \epsilon ^{Mo}_{i,\beta =d_{3z^{2}-r^{2}}}\;\;,\;\;\;\; \Phi _{1}\equiv \epsilon ^{Mo}_{i,\beta =d_{x^{2}-y^{2}}} \;,\;\;\; \Phi _{2}\equiv \epsilon ^{Mo}_{i,\beta =d_{xy}} \end{aligned}$$

Also the parameters $\epsilon ^{S}_{i,\alpha }$ in Eq. 2) make the following matrix presentation for $\epsilon ^{S}_{i}$

4 $$\begin{aligned} \epsilon ^{S}_{i}= & {} \left( \begin{array}{ccc} \Phi _{p}+t_{xx}^{\bot }&{} 0&{}0 \\ 0&{} \Phi _{p}+t_{yy}^{\bot }&{}0 \\ 0&{} 0&{}\Phi _{z}-t_{zz}^{\bot }\\ \end{array} \right) ,\nonumber \\ \Phi _{p}+t_{xx}^{\bot }\equiv & {} \epsilon ^{S}_{i,\alpha =p_{x}^{Sym}}\;\;,\;\;\;\; \Phi _{p}+t_{yy}^{\bot }\equiv \epsilon ^{S}_{i,\alpha =p_{y}^{Sym}} \;,\;\;\; \Phi _{z}-t_{zz}^{\bot }\equiv \epsilon ^{S}_{i,\alpha =p_{z}^{Anti}} \end{aligned}$$

where $t_{xx}^{\bot }=V_{pp\pi }, t_{yy}^{\bot }=V_{pp\pi }, t_{zz}^{\bot }=V_{pp\sigma }$ are the perpendicular hopping amplitudes of electron between S atoms on two different layers. A note is in order here. When we express *p* orbitals in terms of symmetric and antisymmetric forms, i.e. $p^{S}_{x}, p^{S}_{y}, p^{A}_{z}$, , Hamiltonian matrix elements of S orbitals will be different from the 11-band model. Under these transformations that we did, the numerical values of $V_{pp\pi }$ and $V_{pd\pi }$, which are calculated within the density functional theory method, do not change^59,60^. Now we define the suitable expression for each hopping parameter in Eq. 2). All parameters in Eqs. 3,4) is given in Tables 1, 2. According to Fig. 1b, we have three nearest neighbor unit cells with connecting vectors ${\delta }_{l=1,2,3}\equiv \textbf{R}_{j_{l}}-\textbf{R}_{i}$ so that $\textbf{R}_{i}$ is the position vector of *i*th unit cell and $\textbf{R}_{j_{l}}$ denotes the position vector of *l*the nearest neighbor unit cell. In a similar interpretation, we have three next nearest neighbor unit cells with connecting vectors $\textbf{b}_{l=1,2,3}\equiv \textbf{R}_{j_{l}}-\textbf{R}_{i}$ so that $\textbf{R}_{i}$ is the position vector of *i*th unit cell and $\textbf{R}_{j_{l}}$ denotes the position vector of *l*the next nearest neighbor unit cell. Depending the position vector of $j_{l}$ with $l=1,2,3$, we have three different expressions for $t^{Mo-S}_{ij_{l},\alpha \beta }$ in Eq. 2) as $i, j_{l}$ are nearest neighbor lattice sites. The matrix presentation of $t^{Mo-S}_{\delta _{l}}$ with elements of $t^{Mo-S}_{ij_{l},\alpha \beta }\equiv t^{Mo-S}_{\delta _{l},\alpha \beta }$ for different atomic orbital basis $\alpha ,\beta$ is given by

5 $$\begin{aligned} t^{Mo-S}_{\delta _{1}}= & {} \frac{\sqrt{2}}{7\sqrt{7}}\left( \begin{array}{ccc} -9V_{pd\pi }+\sqrt{3}V_{pd\sigma }&{} -V_{pd\sigma }+3\sqrt{3}V_{pd\pi }&{}12V_{pd\pi }+\sqrt{3}V_{pd\sigma } \\ 3V_{pd\sigma }+5\sqrt{3}V_{pd\pi }&{} 9V_{pd\pi }-\sqrt{3}V_{pd\pi }&{}3V_{pd\sigma }-2\sqrt{3}V_{pd\pi } \\ -V_{pd\pi }-3\sqrt{3}V_{pd\sigma }&{}3V_{pd\sigma }+5\sqrt{3}V_{pd\pi }&{}-3\sqrt{3}V_{pd\sigma }+6V_{pd\pi }\\ \end{array} \right) ,\nonumber \\ t^{Mo-S}_{\delta _{2}}= & {} \frac{\sqrt{2}}{7\sqrt{7}}\left( \begin{array}{ccc} 0&{} 2V_{pd\sigma }-6\sqrt{3}V_{pd\pi }&{}12V_{pd\pi }+\sqrt{3}V_{pd\sigma } \\ 0&{} -6V_{pd\pi }-4\sqrt{3}V_{pd\sigma }&{}-6V_{pd\sigma }+4\sqrt{3}V_{pd\pi } \\ 14V_{pd\pi }&{}0&{}0\\ \end{array} \right) ,\nonumber \\ t^{Mo-S}_{\delta _{3}}= & {} \frac{\sqrt{2}}{7\sqrt{7}}\left( \begin{array}{ccc} 9V_{pd\pi }-\sqrt{3}V_{pd\sigma }&{} -V_{pd\sigma }+3\sqrt{3}V_{pd\pi }&{}12V_{pd\pi }+\sqrt{3}V_{pd\sigma } \\ -3V_{pd\sigma }-5\sqrt{3}V_{pd\pi }&{} 9V_{pd\pi }-\sqrt{3}V_{pd\pi }&{}3V_{pd\sigma }-2\sqrt{3}V_{pd\pi } \\ -V_{pd\pi }-3\sqrt{3}V_{pd\sigma }&{}-3V_{pd\sigma }-5\sqrt{3}V_{pd\pi }&{}3\sqrt{3}V_{pd\sigma }-6V_{pd\pi }\\ \end{array} \right) , \end{aligned}$$

as the values of parameters in Eq. 5) are provided in Table. 2. In other hand, the position index $i,j_{l}$ in the matrix elements $t^{Mo-Mo}_{ij_{l},\beta \beta '}$ (according to Eq. 2)) are related to the next nearest neighbor lattice sites. The matrix presentation of $t^{Mo-Mo}_{\textbf{b}_{l}}$ with elements of $t^{Mo-Mo}_{ij_{l},\beta \beta '}\equiv t^{Mo-Mo}_{\textbf{b}_{l},\beta \beta '}$ for different atomic orbital basis $\beta ,\beta '$ is given by

6 $$\begin{aligned} t^{Mo-Mo}_{\textbf{b}_{1}}= & {} \frac{1}{4}\left( \begin{array}{ccc} V_{dd\sigma }+3V_{dd\delta }&{} \frac{\sqrt{3}}{2}\left( -V_{dd\delta }+V_{dd\sigma }\right) &{}-\frac{3}{2}\left( V_{dd\delta }-V_{dd\sigma }\right) \\ \frac{\sqrt{3}}{2}\left( -V_{dd\delta }+V_{dd\sigma }\right) &{} \frac{1}{4}\left( V_{dd\delta }+3V_{dd\sigma }+12V_{dd\pi }\right) &{}\frac{\sqrt{3}}{4}\left( V_{dd\delta }+3V_{dd\sigma }-4V_{dd\pi }\right) \\ -\frac{3}{2}\left( V_{dd\delta }-V_{dd\sigma }\right) &{} \frac{\sqrt{3}}{4}\left( V_{dd\delta }+3V_{dd\sigma }-4 V_{dd\pi }\right) &{}\frac{1}{4}\left( 3V_{dd\delta }+9V_{dd\sigma }+4V_{dd\pi }\right) \\ \end{array} \right) ,\nonumber \\ t^{Mo-Mo}_{\textbf{b}_{2}}= & {} \frac{1}{4}\left( \begin{array}{ccc} V_{dd\sigma }+3V_{dd\delta }&{} \sqrt{3}(V_{dd\delta }-V_{dd\sigma })&{}0 \\ \sqrt{3}(V_{dd\delta }-V_{dd\sigma })&{} V_{dd\delta }+3V_{dd\sigma }&{}0\\ 0 &{} 0 &{}4V_{dd\pi }\\ \end{array} \right) ,\nonumber \\ t^{Mo-Mo}_{\textbf{b}_{3}}= & {} \frac{1}{4}\left( \begin{array}{ccc} V_{dd\sigma }+3V_{dd\delta }&{} \frac{\sqrt{3}}{2}(-V_{dd\delta }+V_{dd\sigma })&{}\frac{3}{2}(V_{dd\delta }-V_{dd\sigma }) \\ \frac{\sqrt{3}}{2}(-V_{dd\delta }+V_{dd\sigma })&{} \frac{1}{4}(V_{dd\delta }+3V_{dd\sigma }+12V_{dd\pi })&{}-\frac{\sqrt{3}}{4}(V_{dd\delta }+3V_{dd\sigma }-4V_{dd\pi })\\ \frac{3}{2}(V_{dd\delta }-V_{dd\sigma })&{} -\frac{\sqrt{3}}{4}(V_{dd\delta 
}+3V_{dd\sigma }-4 V_{dd\pi })&{}\frac{1}{4}(3V_{dd\delta }+9V_{dd\sigma }+4V_{dd\pi })\\ \end{array} \right) , \end{aligned}$$

The matrix presentation of $t^{S-S}_{\textbf{b}_{l}}$ with elements of $t^{Mo-Mo}_{ij_{l},\beta \beta '}\equiv t^{Mo-Mo}_{\textbf{b}_{l},\alpha \alpha '}$ for different atomic orbital basis $\alpha ,\alpha '$ is given by

7 $$\begin{aligned} t^{S-S}_{\textbf{b}_{1}}= & {} \frac{1}{4}\left( \begin{array}{ccc} 3V_{pp\pi }+V_{pp\sigma }&{} \sqrt{3}(V_{pp\pi }-V_{pp\sigma })&{}0 \\ \sqrt{3}(V_{pp\pi }-V_{pp\sigma })&{} V_{pp\pi }+3V_{pp\sigma }&{}0\\ 0&{} 0&{}4V_{pp\pi }\\ \end{array} \right) ,\nonumber \\ t^{S-S}_{\textbf{b}_{2}}= & {} \left( \begin{array}{ccc} V_{pp\sigma }&{} 0&{}0 \\ 0&{} V_{pp\pi }&{}0\\ 0 &{} 0 &{}4V_{pp\pi }\\ \end{array} \right) ,\nonumber \\ t^{S-S}_{\textbf{b}_{3}}= & {} \frac{1}{4}\left( \begin{array}{ccc} 3V_{pp\pi }+V_{pp\sigma }&{} -\sqrt{3}(V_{pp\pi }-V_{pp\sigma })&{}0 \\ -\sqrt{3}(V_{pp\pi }-V_{pp\sigma })&{} V_{pp\pi }+3V_{pp\sigma }&{}0\\ 0&{} 0&{}4V_{pp\pi }\\ \end{array} \right) , \end{aligned}$$

The hopping amplitudes and on-site energies have been calculated in the context of Slater-Koster scheme^26,48,52^ and the values of the parameters are given in Tables 1, 2.

**Table 1:** On-site energy parameter values in Eqs. (3, 4) for monolayer $\text {MoS}_{2}$.

$\Phi _{0}$	$\Phi _{1}$	$\Phi _{2}$	$\Phi _{p}$	$\Phi _{z}$
$-1.512$	0.0	$-3.025$	$-1.276$	$-8.236$

All terms are in units of eV.

**Table 2:** Slater-Koster tight binding parameters in Eqs. (5–7) for monolayer $\text {MoS}_{2}$.

$V_{pp\sigma }$	$V_{pp\pi }$	$V_{pd\sigma }$	$V_{pd\pi }$	$V_{pd\delta }$	$V_{dd\delta }$	$V_{dd\pi }$
0.696	0.278	$-2.619$	$-1.396$	$-0.933$	$-0.442$	0.478

All terms are in units of eV.

Local spin-orbit interaction can be as well included in a suitable way^48^. The large spin-orbit in $\text {MoS}_{2}$ can be approximately understood by intra atomic contribution $H_{SO}\propto \textbf{S}\cdot \textbf{L}$. The final results for nonzero matrix elements of $H_{SO}$ in Hilbert space introduced in Eq. 1) get the following relations

8 $$\begin{aligned} \langle i,d_{xy},s|H_{SO}|i,d_{x^{2}-y^{2}},s\rangle =i\lambda _{Mo}s,\;\;\langle i,p_{y}^{Sym},s|H_{SO}|i,p_{x}^{Sym}, s\rangle =i\frac{\lambda _{S}s}{2}, \end{aligned}$$

where $s=\pm$ indicates the spin angular momentom quantum number of electrons, $\lambda _{Mo}=86$ meV and $\lambda _{S}=52$ meV imply the spin-orbit coupling strength of electron for Mo and S atoms respectively. In Eq. 8), $|i,\beta ,s\rangle$ with $\beta =d_{x^{2}-y^{2}},d_{xy}$ describes the electron quantum state located on Mo atom in lattice site *i* with spin *s* in the atomic orbital labeled by $\beta$. Moreover $|i,\alpha ,s\rangle$ with $\alpha =p_{y}^{Sym},p_{x}^{Sym}$ describes the electron quantum state located on S atom in lattice site *i* with spin *s* in the atomic orbital labeled by $\alpha$. It is worthwile to some comments about only coupling between $d_{x^{2}-y^{2}}$ and $d_{xy}$ in Mo, and $p_{x}$ and $p_{y}$ orbitals are not considered here. Also the $d_{3z^{2}-r^{2}}$ and $p_{z}$ orbitals are not involved in matrix elements of $H_{SO}$. This fact can be demonstrated based on the expansion of $|d_{x^{2}-y^{2}}\rangle$, $|d_{xy}\rangle$ and $|d_{3z^{2}-r^{2}}\rangle$ in terms of eigenvectors of *z*-component of angular momentum $L_{z}$, i.e. $|l,m_{l}\rangle$ in which *l* denotes the quantum number of total angular momentum and $m_{l}=-l,-l+1,...,+l$ refers to quantum number of operator $L_{z}$. We have the following expansions for Mo atoms

9 $$\begin{aligned} |d_{x^{2}-y^{2}}\rangle= & {} \frac{1}{\sqrt{2}}\Big (|l=2,m_{l}=2\rangle +i|l=2,m_{l}=-2\rangle \Big ),\;\;|d_{xy}\rangle = \frac{1}{\sqrt{2}}\Big (|l=2,m_{l}=2\rangle -i|l=2,m_{l}=-2\rangle \Big )\nonumber \\ |d_{3z^{2}-r^{2}}\rangle= & {} |l=2,m_{l}=0\rangle . \end{aligned}$$

Since the spin-orbit coupling model is given by the operator form $H_{SO}\propto \textbf{S}\cdot \textbf{L}\propto S_{z}L_{z}$ and also the matrix elements of operator $L_{z}$ are obtained as $\langle l,m_{l}|L_{z}|l',m_{l'}\rangle =m_{l}\delta _{l,l'}\delta _{m_{l}m_{l'}}$, we can expect that only the non zero matrix elements of $L_{z}$ are $\langle d_{x^{2}-y^{2}}|L_{z}|d_{xy}\rangle$ and $\langle d_{xy}|L_{z}|d_{x^{2}-y^{2}}\rangle$. In fact $|d_{3z^{2}-r^{2}}\rangle$ includes only $|l=2,m_{l}=0\rangle$ in its series expansion in terms of eigenvectors of $L_{z}$, spin-orbit coupling model, i.e. $S_{z}L_{z}$, has no coupling between $d_{3z^{2}-r^{2}}$ and $d_{xy}$. In a similar reason there is no coupling between $d_{3z^{2}-r^{2}}$ and $d_{x^{2}-y^{2}}$. In other words $\langle d_{x^{2}-y^{2}}|L_{z}|d_{3z^{2}-r^{2}}\rangle$ and $\langle d_{3z^{2}-r^{2}}|L_{z}|d_{xy}\rangle$ get zero value. Also we have the following expressions for S atoms

10 $$\begin{aligned} |p_{x}\rangle= & {} \frac{1}{\sqrt{2}}\Big (|l=1,m_{l}=1\rangle +|l=1,m_{l}=-1\rangle \Big ),\;\;|p_{y}\rangle = \frac{1}{\sqrt{2}}\Big (-|l=1,m_{l}=1\rangle +|l=1,m_{l}=-1\rangle \Big )\nonumber \\ |p_{z}\rangle= & {} |l=1,m_{l}=0\rangle . \end{aligned}$$

Because $|p_{z}\rangle$ includes only $|l=1,m_{l}=0\rangle$ in its series expansion in terms of eigenvectors of $L_{z}$, spin-orbit coupling model, i.e. $S_{z}L_{z}$, has no coupling between $p_{z}$ and $p_{x}$. In a similar reason there is no coupling between $p_{z}$ and $p_{y}$. In other words $\langle p_{z}|L_{z}|p_{x}\rangle$ and $\langle p_{z}|L_{z}|p_{y}\rangle$ get zero value.

In the presence of applied magnetic field perpendicular to the plane of $\text {MOS}_{2}$ layer with strength *B*, the Zeeman splitting takes place. Based on matrix form of tight binding model Hamiltonian in Eqs. (3–7) and nonzero matrix elements of spin-orbit coupling model Hamiltonian, the model Hamiltonian for electrons with spin quantum number $s=\pm$ can be expressed in a more compact form once written in the k space:

11 $$\begin{aligned} H=H^{TB}+H_{SO}= & {} \left( \begin{array}{cc} H^{Mo-Mo}(\textbf{k})&{} H^{Mo-S}(\textbf{k}) \\ (H^{Mo-S}(\textbf{k}))^{\dag }&{} H^{S-S}(\textbf{k})\\ \end{array} \right) ,\nonumber \\ H^{Mo-Mo}(\textbf{k})= & {} \epsilon ^{Mo}_{i}+\left( \begin{array}{ccc} 0&{} 0&{}0 \\ 0&{} 0 &{}-i\lambda _{Mo}s\\ 0&{} i\lambda _{Mo}s &{}0\\ \end{array} \right) +2\sum _{j=1,2,3}t^{Mo-Mo}_{\textbf{b}_{j}}cos(\textbf{k}\cdot \textbf{b}_{j})-sg\mu _{B}B\textbf{1},\nonumber \\ H^{S-S}(\textbf{k})= & {} \epsilon ^{S}_{i}+\left( \begin{array}{ccc} 0&{} -\frac{i\lambda _{Mo}s}{2}&{}0 \\ \frac{i\lambda _{Mo}s}{2}&{} 0 &{}0\\ 0&{} 0 &{}0\\ \end{array} \right) +2\sum _{j=1,2,3}t^{S-S}_{\textbf{b}_{j}}cos(\textbf{k}\cdot \textbf{b}_{j})-sg\mu _{B}B\textbf{1}\nonumber \\ H^{Mo-S}(\textbf{k})= & {} \sum _{j=1,2,3}t^{Mo-S}_{{\delta }_{j}}e^{i\textbf{k}\cdot {\delta }_{j}}, \end{aligned}$$

so that $g\approx 2.0$ is the gyromagnetic constant and $\mu _{B}$ describes the Bohr magneton constant. Also in above equation, $\textbf{1}$ implies 3$\times$3 unit matrix. Moreover the nearest ($\delta _{j}$) and the next nearest ($\textbf{b}_{j}$) neighbor vectors have been shown in Fig. 1a and can be written as

12 $$\begin{aligned} {\delta }_{j=1}= & {} b_{0}\Big (-\frac{\sqrt{3}}{2}\cos(\theta _{B}),-\frac{1}{2}\cos(\theta _{B}),\sin(\theta _{B})\Big )\nonumber \\ {\delta }_{j=2}= & {} b_{0}\Big (0,\cos(\theta _{B}),\sin(\theta _{B})\Big )\nonumber \\ {\delta }_{j=3}= & {} b_{0}\Big (\frac{\sqrt{3}}{2}\cos(\theta _{B}),-\frac{1}{2}\cos(\theta _{B}),\sin(\theta _{B})\Big )\nonumber \\ \textbf{b}_{j=1}= & {} b(1,0),\;\;\textbf{b}_{j=2}=b\Big (-\frac{1}{2},\frac{\sqrt{3}}{2}\Big ),\;\;\textbf{b}_{j=3}=b\Big (-\frac{1}{2},-\frac{\sqrt{3}}{2}\Big ), \end{aligned}$$

that $b_{0}=2.41 A^{0}$ denotes the interatomic distance between Mo and S atoms. $b=3.16 A^{0}$ is the binding distance for Mo-Mo and $\theta _{B}=acos(\sqrt{\frac{4}{7}})$.

The Slater-Koster tight-binding method has been applied when lattice deformations, like strain, are considered. Hence the effect of strain is driven by the dependence of tight-binding amplitudes on the interatomic distance. In the present study, the varying the interatomic bond length, as a result of applied strain, leads to the strain effects. Up to the linear order, the hopping matrixes in Eqs. (5,6,7) have been modified in the presence of strain effects as^61,62^

13 $$\begin{aligned} t^{Mo-S}_{{\widetilde{\delta }}_{i}}= & {} t^{Mo-S}_{\delta _{i}}\left( 1-\beta _{2}\left( \frac{{\widetilde{\delta }}_{i}}{\delta _{i}}-1\right) \right) ,\;\; t^{S-S}_{\widetilde{\textbf{b}}_{i}}=t^{S-S}_{\textbf{b}_{i}}\left( 1-\beta _{1}\left( \frac{{\widetilde{b}}_{i}}{{b}_{i}}-1\right) \right) \nonumber \\ t^{Mo-Mo}_{\widetilde{\textbf{b}}_{i}}= & {} t^{Mo-Mo}_{\textbf{b}_{i}}\left( 1-\beta _{3}\left( \frac{{\widetilde{b}}_{i}}{{b}_{i}}-1\right) \right) , \end{aligned}$$

where $\delta _{i}=\sqrt{\frac{7}{12}}b$ with $i=1,2,3$ is the distance in the equilibrium positions between two nearest neighbor Mo and S atoms (Mo-S bonding length), ${\widetilde{\delta }}_{i}$ with $i=1,2,3$ describes the distance in the presence of strain. $b_{i}=b$ with $i=1,2,3$ denotes the distance in the equilibrium positions between two next nearest neighbor atoms (in-plane Mo-Mo and S-S bonding lengths). Moreover ${\widetilde{b}}_{i}$ is the distance between next nearest neighbor atoms in the presence strain. According to the definition of the electron-phonon coupling constant, the parameters $\beta _{1},\beta _{2}, \beta _{3}$ are obtained for *pp*, *pd*, *dd* hybridization, respectively. The values of these parameters are $\beta _{1}=3,\beta _{2}=4, \beta _{3}=5$. In order to study the effects of strain on the electronic properties of $\text {MoS}_{2}$, it is necessary to express the deformed bond lengths in terms of strain parameter and equilibrium bond lengths. We assume a general form of homogenous deformation with a large wavelength inhomogeneous strain. This strain can be considered as a powerful tool to locally manipulate the electronic structure of two-dimensional electronic systems even in atomic scale. To consider such deformation, we use the following relations for the bond lengths, i.e. $\widetilde{\textbf{b}}_{i}$ with $i=1,2,3$ and ${\widetilde{\delta }}_{i}$ with $i=1,2,3$

14 $$\begin{aligned} {\widetilde{\delta }}_{i=1}= & {} b\sqrt{\left( \frac{1}{2}+\frac{\varepsilon _{xx}}{2}\right) ^{2}+\left( \frac{1}{2\sqrt{3}}+ \frac{\varepsilon _{yy}}{2\sqrt{3}}\right) ^{2}+\frac{1}{4}}\nonumber \\ {\widetilde{\delta }}_{i=2}= & {} b\sqrt{\left( \frac{1}{\sqrt{3}}+\frac{\varepsilon _{yy}}{\sqrt{3}}\right) ^{2}+\frac{1}{4}},\;\; {\widetilde{\delta }}_{i=3}={\widetilde{\delta }}_{i=1}\nonumber \\ {\widetilde{b}}_{i=1}= & {} b\sqrt{\left( \frac{1}{2}+\frac{\varepsilon _{xx}}{2}\right) ^{2}+\left( \frac{\sqrt{3}}{2}+\frac{\sqrt{3}\varepsilon _{yy}}{2}\right) ^{2}},\;\; {\widetilde{b}}_{i=2}=b(1+\varepsilon _{xx}),\;\; {\widetilde{b}}_{i=3}={\widetilde{b}}_{i=1}. \end{aligned}$$

For homogenous biaxial strain we consider $\varepsilon _{xx}=\varepsilon _{yy}=\varepsilon$. Under applying the uniaxial strain to the $\text {MoS}_{2}$ plane, we have $\varepsilon _{xx}=-0.125\varepsilon$ and $\varepsilon _{yy}=\varepsilon$ so that $\varepsilon$ describes the strain parameter^54^. Using the relations in Eq. 14), the deformed bond lengths, i.e. ${\widetilde{\delta }}_{i}$ and ${\widetilde{b}}_{i}$, are expressed in terms of strain parameter $\varepsilon$ and undeformed bond length *b*. Under substitutions $t^{Mo-S}_{\delta _{i}}\longrightarrow t^{Mo-S}_{{\widetilde{\delta }}_{i}}$, $t^{S-S}_{\textbf{b}_{i}}\longrightarrow t^{S-S}_{\widetilde{\textbf{b}}_{i}}$ and $t^{Mo-Mo}_{\textbf{b}_{i}}\longrightarrow t^{Mo-Mo}_{\widetilde{\textbf{b}}_{i}}$ in matrix representation of Hamiltonian in Eq. 11), we obtain the effects of strain on electronic properties of $\text {MoS}_{2}$ layer.

Using the Hamiltonian in Eq. 11) with redefined hopping parameters in Eq. 13), the band structure of electrons with spin *s* of strained $\text {MoS}_{2}$ layer has been found by solving equation $det\Big (H-E^{s}_{\eta }(\textbf{k})\textbf{1}\Big )=0$ where $\eta =1,2,...,6$ denotes the quantum number of band structure and $\textbf{1}$ introduces 3$\times$3 unit matrix. In the presence of magnetic field perpendicular to the plane of $\text {MoS}_{2}$ layer, the final results for electronic band structure of $\text {MoS}_{2}$ for spin *s* are numerically found and are named by $E^{s}_{\eta }(\textbf{k})$.

Using band energy spectrum, the Hamiltonian in Eq. 11) can be rewritten by

15 $$\begin{aligned} H=\sum _{\textbf{k},s,\eta } E^{s}_{\eta }(\textbf{k})c^{\dag s}_{\eta ,\textbf{k}}c^{s}_{\eta ,\textbf{k}}, \end{aligned}$$

where $c^{s}_{\eta ,\textbf{k}}$ defines the creation operator of electron with spin *s* in band index $\eta$ at wave vector $\textbf{k}$. The electronic Green’s function can be defined using the Hamiltonian in Eq. (15) as following expression

16 $$\begin{aligned} G^{s}_{\eta }(\textbf{k},\tau )=-\langle T_{\tau } c^{s}_{\eta ,\textbf{k}}(\tau )c^{\dag s}_{\eta ,\textbf{k}}(0)\rangle , \end{aligned}$$

where $\tau$ is imaginary time. Using the model Hamiltonian in Eq. (15), the Fourier transformations of Green’s function is given by

17 $$\begin{aligned} G^{s}_{\eta }(\textbf{k},i\omega _{n})=\int ^{1/k_{B}T}_{0}d\tau e^{i\omega _{n}\tau } G^{s}_{\eta }(\textbf{k},\tau )=\frac{1}{i\omega _{n}-E^{s}_{\eta }(\textbf{k})}. \end{aligned}$$

Here $\omega _{n}=(2n+1)\pi k_{B}T$ denotes the fermionic Matsubara frequency in which *T* is equilibrium temperature. Total electronic density of states of $\text {MoS}_{2}$ due biaxial strains and under applying external magnetic field can be obtained by electronic band structure as

18 $$\begin{aligned} Dos(E)=-\frac{1}{2N}Im\sum _{\textbf{k},s,\eta }\frac{1}{E-E^{s}_{\eta }(\textbf{k})+i0^{+}}. \end{aligned}$$

Summation over wave vectors have been performed into first Brillouin zone of honeycomb lattice. The density of states includes prominent asymmetric peaks due to the band edge of parabolic subbands. The peaks positions arise from the band edge state energies and the density of states heights are proportional to inverse square root of the subband curvature and band degeneracy. For determining the chemical potential, $\mu$, we use the relation between concentration of electrons ($n_{e}$) and chemical potential. This relation is given by

19 $$\begin{aligned} n_{e}=\frac{1}{4N}\sum _{\textbf{k},\eta ,s}\frac{1}{e^{E^{s}_{\eta }(\textbf{k})/k_{B}T}+1}. \end{aligned}$$

Based on the values of electronic concentration $n_{e}$, the chemical potential, $\mu$, can be obtained by means Eq. (19).

## Optical absorption of single layer $\text {MoS}_{2}$

The optical conductivity is obtained as the response of the electrical current ($\mathbf{{J}}_{e}$) to an external electrical field. Imposing the continuity equation for the charge density $\rho$, i.e. $\frac{\partial }{\partial t}\rho +\nabla \cdot {\textbf {J}}_{e}=0$, the explicit form of the electrical current operator can be calculated. This calculation has been done for a bilinear Hamiltonian describing Fermionic system^63^ and we can exploit this result for model Hamiltonian in Eq. (15). The operator form of electrical current operator $\textbf{J}_{e}$ for itinerant electrons of $\text {MoS}_{2}$ layer is given by

20 $$\begin{aligned} \textbf{J}_{e}=\sum _{\textbf{k},s,\eta } \textbf{v}_{s,\eta }(\textbf{k})c^{\dag s}_{\eta ,\textbf{k}}c^{s}_{\eta ,\textbf{k}}, \end{aligned}$$

so that $\textbf{v}_{s,\eta }(\textbf{k})=\mathbf {\nabla }_\textbf{k}E^{s}_{\eta }(\textbf{k})$ denotes the group velocity of electrons with spin *s* at band structure $\eta$. The linear response theory is implemented to obtain the optical conductivity under the assumption of a low dynamical electric field (as a perturbing field). The Kubo formula gives the transport coefficient $\sigma (\omega )$ in terms of a correlation function of electrical current operators

21 $$\begin{aligned}{} & {} \sigma (\omega )=\frac{1}{\omega }Im\Big (i\int _{-\infty }^{+\infty }dt e^{i\omega t}\theta (t)\langle [J^{x}_{e}(t),J^{x}_{e}(0)]\rangle \Big ) \nonumber \\ {}= & {} \frac{1}{\omega }Im\Big (\lim _{i\omega _{n}\longrightarrow \omega +i0^{+}} \int ^{1/k_{B}T}_{0}d\tau e^{i\omega _{n}\tau }\langle T_{\tau }(J^{x}_{e}(\tau )J^{x}_{e}(0))\rangle \Big ), \end{aligned}$$

where it is assumed that electrical current flows along zigzag direction, i.e. *x* direction in Fig. 1,. By implementing Wick’s theorem, we can calculate the correlation function between current operators in Eq. (21) as Applying the Wick’s theorem leads to the following expression for energy current correlation function as

22 $$\begin{aligned} \langle T_{\tau }(J^{x}_{e}(\tau )J^{x}_{e}(0))\rangle =\sum _{\textbf{k},\eta ,s}(v^{x}_{s,\eta }(\textbf{k}))^{2} G^{s}_{\eta }(\textbf{k},\tau )G^{s}_{\eta }(\textbf{k},-\tau ). \end{aligned}$$

By substituting Eq. (22) into Eq. (21) and using Fourier transformation of bosonic Green’s function, i.e. $G^{\sigma }_{\eta }(\textbf{k},\tau )=k_{B}T\sum _{m}e^{-i\omega _{m}\tau }G^{\sigma }_{\eta }(\textbf{k},i\omega _{m})$, optical conductivity $\sigma (\omega )$ can be expressed in terms of bosonic Green’s function as

23 $$\begin{aligned} \sigma (\omega ) =\frac{k_{B}T}{\omega }Im\Big (\lim _{i\omega _{n}\longrightarrow \omega +i0^{+}} \sum _{\textbf{k},\eta ,s}\sum _{m}(v^{x}_{s,\eta }(\textbf{k}))^{2} G_{\eta }^{s}(\textbf{k},i\omega _{m})G^{s}_{\eta }(\textbf{k},i\omega _{n}+i\omega _{m})\Big ). \end{aligned}$$

According to the Lehmann representation^63^, the imaginary part of retarded Green’s function and Matsubara form of Green’s function are related to each other as

24 $$\begin{aligned} G^{s}_{\eta }(\textbf{k},i\omega _{m})=\int ^{+\infty }_{-\infty }\frac{d\epsilon }{2\pi }\frac{-2 Im\Big (G^{s}_{\eta } (\textbf{k},\epsilon +i0^{+})\Big )}{i\omega _{m}-\epsilon }, \end{aligned}$$

Using Lehmann representation, the expression for optical conductivity $\sigma (\omega )$ in Eq. (23) is given by

25 $$\begin{aligned} \sigma (\omega )= & {} \frac{k_{B}T}{\omega }Im\Big (\lim _{i\omega _{n}\longrightarrow \omega +i0^{+}} \sum _{\textbf{k},\eta }\sum _{s}\sum _{m}\int ^{+\infty }_{-\infty }\frac{d\epsilon }{2\pi }\int ^{+\infty }_{-\infty }\frac{d\epsilon '}{2\pi } \Big [(v^{x}_{s,\eta }(\textbf{k}))^{2} 2 Im\Big (G^{s}_{\eta } (\textbf{k},\epsilon +i0^{+})\Big )\nonumber \\ {}\times & {} 2 Im\Big (G^{s}_{\eta } (\textbf{k},\epsilon '+i0^{+})\Big ) \Big ]\frac{1}{i\omega _{m}-\epsilon } \frac{1}{i\omega _{n}+i\omega _{m}-\epsilon '}\Big ). \end{aligned}$$

After summation over Matsubara’s fermionic frequency $\omega _{m}$ and some algebraic calculations, the final result form for optical conductivity of $\text {MoS}_{2}$ layer is given by

26 $$\begin{aligned} \sigma (\omega )=\frac{1}{\omega }\Big \{ \sum _{\textbf{k},\eta }\sum _{s}\int ^{+\infty }_{-\infty }\frac{d\epsilon }{2\pi } \Big [(v^{x}_{s,\eta }(\textbf{k}))^{2} 2 Im\Big (G^{s}_{\eta } (\textbf{k},\epsilon +i0^{+})\Big )2 Im\Big (G^{s}_{\eta } (\textbf{k},\epsilon +\omega +i0^{+})\Big ) \Big ]\frac{n_{F}(\epsilon +\omega )-n_{F}(\epsilon )}{\omega }\Big \}, \end{aligned}$$

where $n_{F}(x)=\frac{1}{e^{x/k_{B}T}+1}$ is the Fermi-Dirac distribution function and *T* denotes the equilibrium temperature. Substituting electronic Green’s function presented in Eq. (17) into Eq. (26) and performing the numerical integration over wave vector through first Brillouin zone, the results of optical absorption in terms of photon frequency $\omega$ have been obtained. Here, the contribution of both inter and intra band transitions on the optical conductivity in Eq. (26) has been considered. The imaginary part of dielectric function corresponding to the rate of photon absorption by gapped graphene is related to dynamical electrical conductivity via

27 $$\begin{aligned} Im(\epsilon (\omega ))=\frac{4\pi }{\omega }Re(\sigma (\omega )). \end{aligned}$$

The numerical results of optical absorption of single layer $\text {MoS}_{2}$ in the presence of strains and magnetic field are presented in the next section.

**Figure 2:**
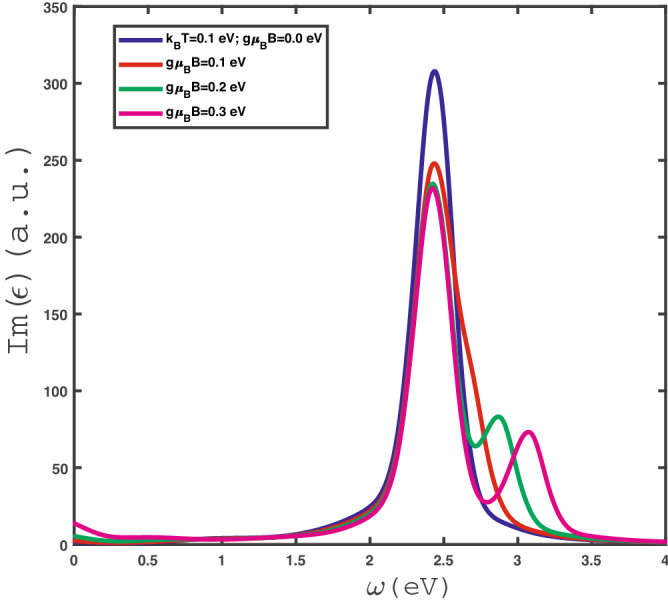
Optical absorption, $Im(\epsilon )$, of undoped $\text {MoS}_{2}$ layer as a function of photon frequency in the absence of homogenous strain , i.e. $\varepsilon _{x}=\varepsilon _{y}=0$, for different values of applied magnetic field $g\mu _{B}B$ at fixed temperature $k_{B}T=0.1$ eV.

**Figure 3:**
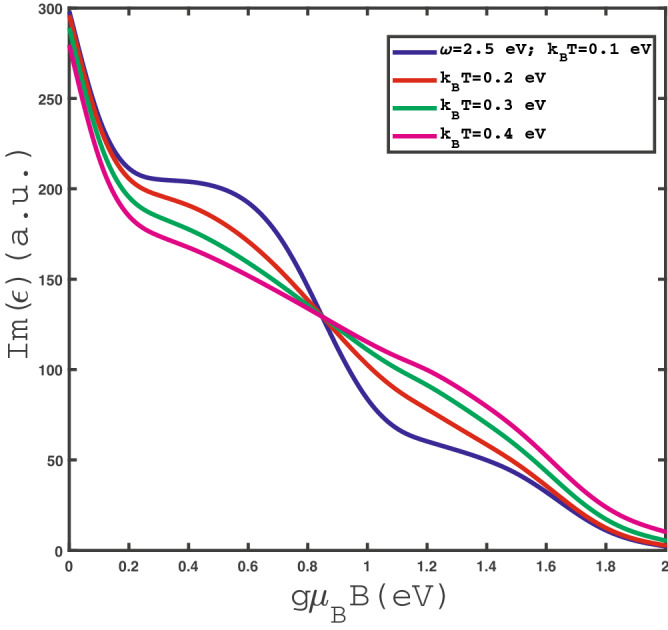
Optical absorption, $Im(\epsilon )$, of undoped $\text {MoS}_{2}$ layer as a function of magnetic field $g\mu _{B}B$ at fixed photon frequency $\omega =2.5$ eV for different values of temperature in the absence of strain.

**Figure 4:**
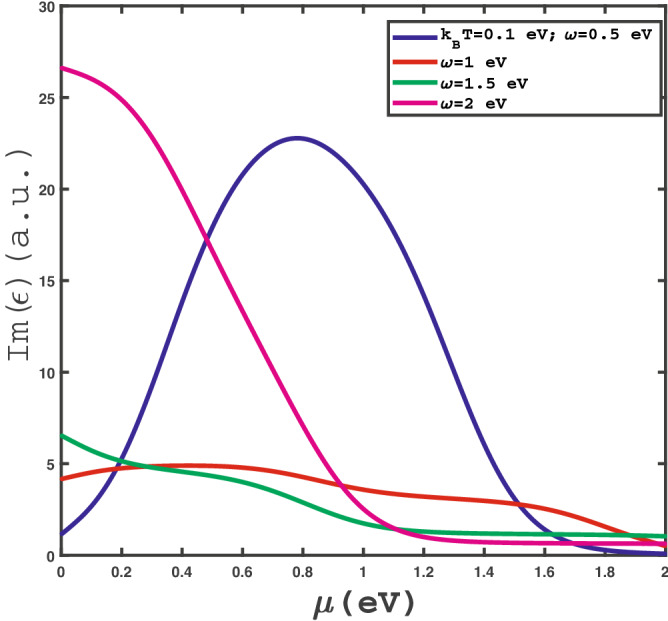
Optical absorption, $Im(\epsilon )$, of doped $\text {MoS}_{2}$ layer as a function of chemical potential $\mu$ at fixed temperature $k_{B}T=0.1$ eV for different values of frequency $\omega$ in the absence of strain.

**Figure 5:**
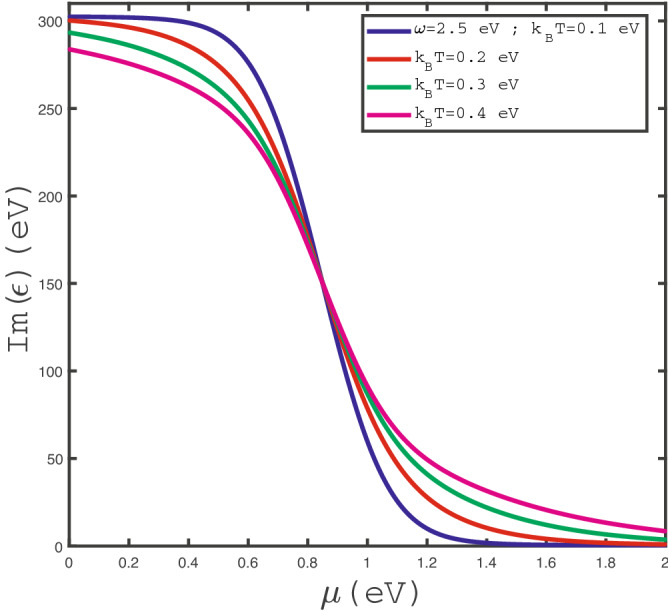
Optical absorption, $Im(\epsilon )$, of doped $\text {MoS}_{2}$ layer as a function of chemical potential $\mu$ at fixed frequency $\omega =2.5$ eV for different values of temperature, namely $k_{B}T=0.1~\text {eV},0.2~\text {eV},0.3~\text {eV},0.4~\text {eV}$, in the absence of strain.

## Discussion and conclusion

In this section, we have presented the numerical results of optical absorption rate of electromagnetic waves in $\text {MoS}_{2}$ monolayer in the presence of magnetic field, spin-orbit coupling and homogenous strain effects. We investigate the frequency dependence of optical conductivity $Im(\epsilon (\omega ))$ of $\text {MoS}_{2}$ layer due to variation of physical parameters. Using dimensionless homogenous strain parameter $\varepsilon$, the amounts of redefined hopping amplitudes of $\text {MoS}_{2}$ layer, i.e. $t^{Mo-S}_{{\widetilde{\delta }}_{i}}, t^{S-S}_{\widetilde{\textbf{b}}_{i}}, t^{Mo-Mo}_{\widetilde{\textbf{b}}_{i}}$, have been obtained by Eqs. (14, 13). With redefination of hopping amplitudes of strained $\text {MoS}_{2}$ layer in matrix $\text {MoS}_{2}$= representation of Hamiltonian in Eq. (11), we can obtain the band structure of $\text {MoS}_{2}$ layer in the presence of strain, spin-orbit coupling effects and external applied magnetic field. Using the electronic band structure of $\text {MoS}_{2}$ monolayer, we can obtain the electronic Green’s function in Eq. (17). It should be noted that the variation of chemical potential $\mu$ leads to various electronic concentration according to Eq. (19). Afterwards density of states, dynamical electrical conductivity $\sigma (\omega )$ are found by substitution of Green’s function into Eq. (26) so that optical absorption rate can be found by Eq. (27). In our numerical results we assume the polarization of electric field of electromagnetic wave is along the zigzag direction according to Fig. 1. Both inter and intra band transitions contribute to the results of optical properties of $\text {MoS}_{2}$ monolayer. The distance between two next nearest neighbor atoms in the atomic structure of the $\text {MoS}_{2}$ layer is assumed to be $b=1$.

**Figure 6:**
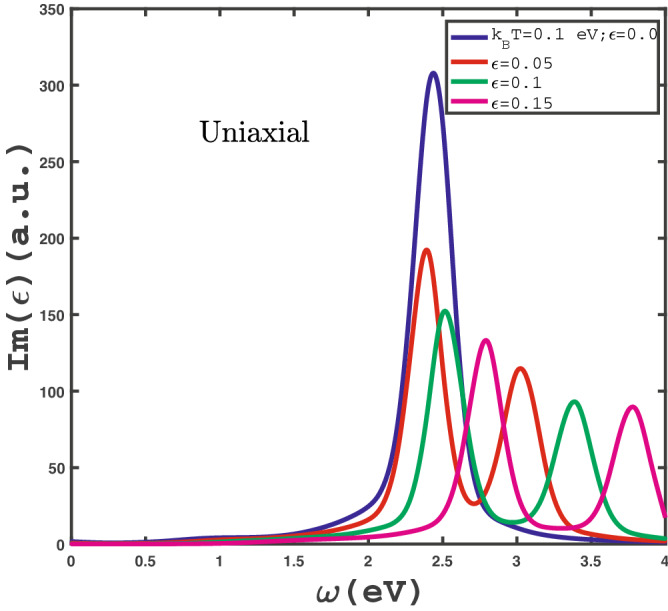
Optical absorption, $Im(\epsilon )$, of undoped $\text {MoS}_{2}$ layer as a function of photon frequency in the presence of positive uniaxial strain for different strain parameter , namely $\varepsilon =0.0,0.05,0.1,0.15$, in the absence of magnetic field at fixed temperature $k_{B}T=0.1$ eV.

The frequency dependence of optical absorption of single layer undoped $\text {MoS}_{2}$ in the absence of strain for different values of magnetic field has been shown in Fig. 2. The temperature has been fixed at amount $k_{B}T=0.1$ eV. Based on this figure, it is clearly observed that the Drude weight value increases with magnetic field so that the Drude weight for $g\mu _{B}B=0.3$ eV gets around normalized value 15. The origin of this Drude weight or the zero frequency limit of optical absorption comes from intraband transition of electrons due to classical behavior of them in this limit. In other hand the peak in optical absorption for all amounts of magnetic field appears at finite frequency $\omega \approx 2.5$ eV. The height of this peak decreases with magnetic field. Such peak arises from interband transition of electrons. An additional peak appears in optical conductivity for magnetic fields $g\mu _{B}B=0.2,\;0.3$ eV. This additional peak is located at higher frequencies rather than first peak position. The frequency position of second peak in optical conductivity for $g\mu _{B}B=0.2$ eV ($g\mu _{B}B=0.3$ eV) is found around $\omega \approx 2.85$ eV ($\omega \approx 3.1$ eV). Also the optical absorption decreases with frequency in the region $\omega >3.25$ eV.

Figure 3 shows the dependence of optical absorption $Im(\epsilon )$ on magnetic field $g\mu _{B}B$ for different values of temperature $k_{B}T$ at fixed frequency $\omega =2.5$ eV in the absence of any type of strain, i.e. $\varepsilon =0.0$. A monotonic decreasing behavior for magnetic field dependence of optical absorption for each value of temperature is clearly observed. It can be understood from this point that increasing magnetic field leads to enhance the band gap in density of states and consequently optical absorption of single layer $\text {MoS}_{2}$ decreases with magnetic field. At fixed magnetic field in the region $g\mu _{B}B>0.8$ eV, the increase of temperature leads to enhance the transition rate of electrons so that optical absorption rises in this magnetic field region. Also Fig. 3 implies at fixed magnetic field in the region $g\mu _{B}B<0.8$ eV, the increase of temperature reduces the optical absorption of $\text {MoS}_{2}$ layer due to scattering rate of electrons. Moreover the optical absorption curves for $k_{B}T=0.05~\text {eV},\;0.07~\text {eV}, \; 0.1~\text {eV}$ fall on each other at magnetic fields above 2.0 eV.

The behavior of chemical potential dependence of optical conductivity of doped $\text {MoS}_{2}$ layer for different photon frequencies, namely $\omega =0.5,1.0,1.5,2.0$, has been shown in Fig. 4. The temperature has been fixed at $k_{B}T=0.1$ eV and any type strain parameter value is assumed to be zero. This figure implies that there is a peak in chemical potential dependence of $Im\varepsilon (\omega )$ for frequency value $\omega =0.5$. At chemical potential values $\mu >0.8$ eV for $\omega =0.5$, the increase of chemical potential leads to enhance the electronic concentration so that the scattering rate between electrons rises and consequently $Im(\epsilon )$ reduces with $\mu$ in this region. In other hand at chemical potentials below 0.8 eV, the increase of $\mu$ causes to the transition rate of electrons from ground state to excited ones. This fact denotes the increase of optical absorption with chemical potential in the region $\mu <0.8$ at $\omega =0.5$. However there is no peak in optical absorption for the other frequency values, i.e. $\omega =1.0,1.5,2.0$.

**Figure 7:**
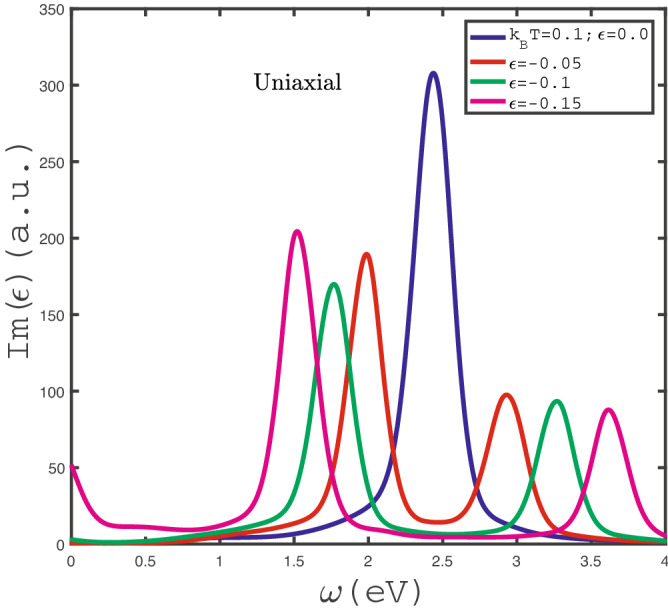
Optical absorption, $Im(\epsilon )$, of undoped $\text {MoS}_{2}$ layer as a function of photon frequency in the presence of negative uniaxial strain for different strain parameter, namely $\varepsilon =0.0,-0.05,-0.1,-0.15$, in the absence of magnetic field at fixed temperature $k_{B}T=0.1$ eV.

We have also studied the effects of temperature on chemical potential dependence of $Im(\epsilon (\omega ))$. In Fig. 5, we have presented the behavior of optical absorption of doped $\text {MoS}_{2}$ layer in terms of $\mu$ in the absence of any strain parameter for different values of temperature. The photon frequency has been fixed at $\omega =2.5$ eV. This plot indicates a monotonic decreasing behavior for each value of temperature. It can be understood from the fact that the increase of chemical potential leads to enhance the electronic concentration. Thus the scattering rate between electrons rises which reduces the optical conductivity. Another feature is pronounced in this figure. For fixed chemical potential below 0.8 eV, $Im(\epsilon (\omega ))$ decreases with temperature according to Fig. 5. Higher temperatures cause more scattering of electrons which reduces the optical conductivity. However for chemical potentials above 0.8 eV, the increase of temperature leads to enhance the transition rate of electrons between quantum energy levels. This fact implies optical conductivity increases with temperature in the chemical potential region above 0.8 eV.

We have studied the effect of in-plane uniaxial strain along armchair direction with strain parameter $\varepsilon$ on frequency dependence of $Im(\epsilon )$ for undoped single layer $\text {MoS}_{2}$ structure in Fig. 6. In this figure, the effects of different positive uniaxial strain, namely $\varepsilon =0.0,0.05,0.1,0.15$, on behavior of $Im(\epsilon )$ of $\text {MoS}_{2}$ layer as a function of frequency have been shown. The applied longitudinal magnetic field is assumed to be zero and temperature has been fixed at $k_{B}T=0.1$ eV. In the absence of strain parameter, a peak appears at finite frequency $\omega =2.4$ eV due to interband transition effects of electrons. For finite non zero strain parameter, two peaks in optical absorption appear at finite frequencies. According to Fig. 6, the intensity of optical absorption at peak frequency position decreases with uniaxial strain. Also the distance between two peaks in $Im(\epsilon )$ at finite uniaxial strain parameter increases with $\varepsilon$ as shown in Fig. 6. Moreover the figure indicates that the optical absorption vanishes in frequency region $\omega <1.5$ for all values of $\varepsilon$. Also there is no Drude weight at zero frequency limit of optical absorption for all uniaxial strains due to band gap in excitation spectrum of the electronic band structure. In fact, the intraband transition in the presence of uniaxial strain has no considerable contribution to the electronic transition and consequently there is no Drude weight in optical absorption of $\text {MoS}_{2}$ under these conditions. This fact shows that $\text {MoS}_{2}$ in the presence of uniaxial strain and in the absence of magnetic field behaves as a metal.

The behaviors of frequency dependence of optical absorption of undoped $\text {MoS}_{2}$ layer for different negative uniaxial strains have been presented in Fig. 7. The temperature and magnetic field have been assumed to be $k_{B}T=0.1$ eV and $g\mu _{B}B=0.0$ eV. There are two peaks in optical absorption at finite frequency for each non zero strain parameter so that the distance between peaks increases with absolute value of $\varepsilon$. Only for $\varepsilon =-0.15$, optical absorption gets the non zero value at zero limit frequency. In other words, there is non zero Drude weight in optical absorption for $\varepsilon =-0.15$. Such fact demonstrates that intraband electronic transition contributes to the optical absorption and consequently the system behaves as a metal. $\text {MoS}_{2}$ layer under negative biaxial strain behaves as a transparent media at frequencies $\omega >4.0$ eV so that optical absorption gets the zero value in this frequency region. Also $Im(\epsilon )$ vanishes for $\varepsilon =0.0,-0.05,-0.1$ at frequencies $\omega <0.7$ eV.

**Figure 8:**
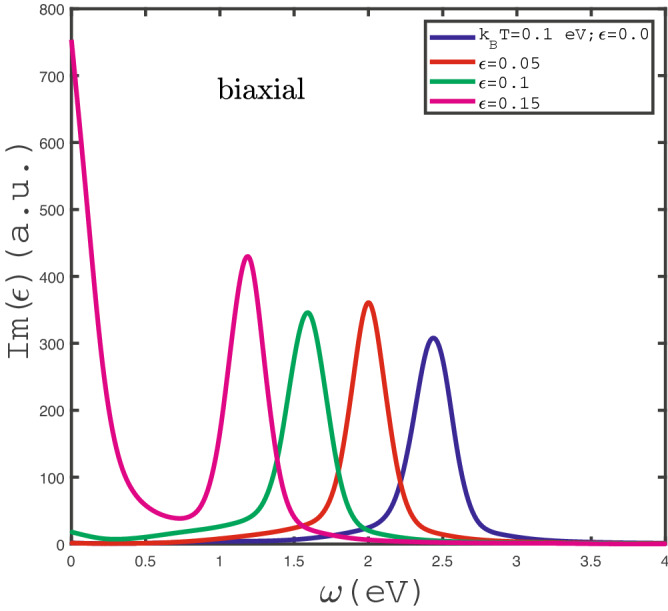
Optical absorption, $Im(\epsilon )$, of undoped $\text {MoS}_{2}$ layer as a function of photon frequency in the presence of positive biaxial strain for different strain parameter , namely $\varepsilon _{x}=\varepsilon _{y}=\varepsilon =0.0,0.05,0.1,0.15$, in the absence of magnetic field at fixed temperature $k_{B}T=0.1$ eV.

The effects of in-plane biaxial strain $\varepsilon _{xx}=\varepsilon _{yy}=\varepsilon$ with positive sign on behaviors of optical absorption have been studied in Fig. 8. We have plotted the frequency dependence of $Im(\epsilon (\omega ))$ for different values of positive in-plane biaxial strain $\varepsilon$ in the absence of magnetic field and temperature $k_{B}T=0.1$ eV in Fig. 8. The intraband band transition contributes to the optical absorption at zero frequency limit for $\varepsilon =0.15$ so that Drude weight takes the remarkable value for this strain amount. In addition to the Drude weight in optical absorption, the finite frequency peaks in optical absorption curves are clearly observed. Such finite frequency peaks in optical absorption arises from electronic interband transitions. The frequency position of peak in optical conductivity moves to lower amounts with $\varepsilon$. This can be understood from this fact that the increase of $\varepsilon$ causes the decrease of band gap in density of states. Thus the peak in $Im(\epsilon (\omega ))$ appears at lower frequency with increase of strain parameter $\varepsilon$. Another novel feature in $Im(\epsilon (\omega ))$ is pronounced in Fig. 8. The optical absorption gets the zero value at frequencies $\omega <1.0$ eV for $\varepsilon =0.0,0.05$. The metallic property of $\text {MoS}_{2}$ layer in the presence of strain value $\varepsilon =0.15$ is considerable since the Drude weight in optical conductivity for strain parameter $\varepsilon =0.15$ gets higher value in comparison with the other values of biaxial strain parameters.

**Figure 9:**
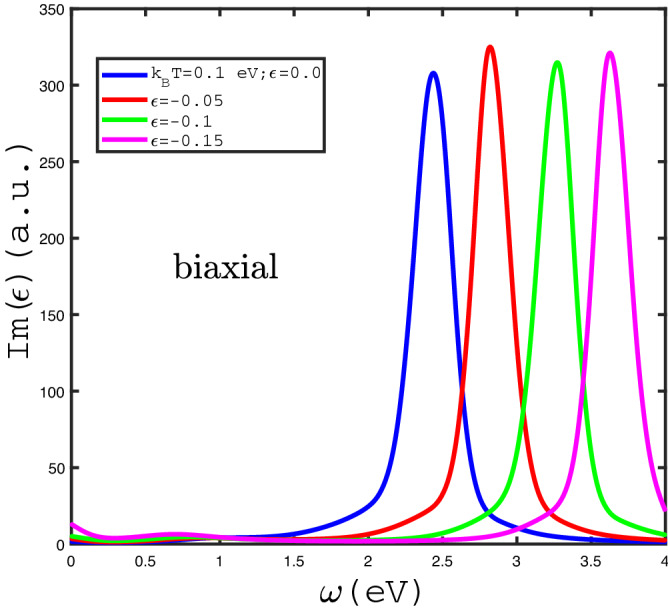
Optical absorption, $Im(\epsilon )$, of undoped $\text {MoS}_{2}$ layer as a function of photon frequency in the presence of negative biaxial strain for different strain parameter , namely $\varepsilon _{x}=\varepsilon _{y}=\varepsilon =0.0,-0.05,-0.1,-0.15$, in the absence of magnetic field at fixed temperature $k_{B}T=0.1$ eV.

The behaviors of frequency dependence of optical absorption of undoped $\text {MoS}_{2}$ layer for different negative biaxial strains have been presented in Fig. 9. The temperature and magnetic field have been assumed to be $k_{B}T=0.1$ eV and $g\mu _{B}B=0.0$ eV. There is a finite frequency peak in optical absorption for each value of strain parameter so that the peak position tends to higher frequency with absolute value of $\varepsilon$. According to Fig. 9, Drude weight of $\text {MoS}_{2}$ layer gets a very low value for all amounts in-plane biaxial strain. It can be justified from this point that intraband electronic transition gives no considerable contribution to the optical absorption and consequently the system behaves as a insulator. $\text {MoS}_{2}$ layer under negative biaxial strain behaves as a transparent media at frequencies $\omega >4.0$ eV and $\omega <1.5$ since $Im(\epsilon )$ gets the zero value in these frequency regions.

**Figure 10:**
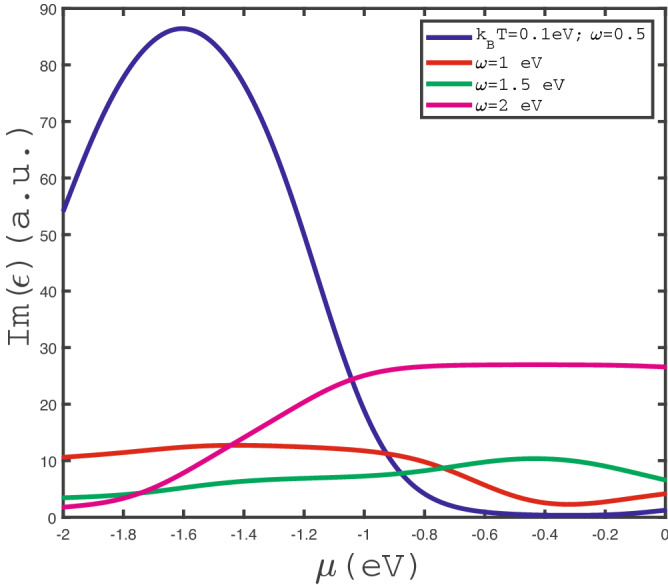
Optical absorption, $Im(\epsilon )$, of doped $\text {MoS}_{2}$ layer as a function of chemical potential $\mu$ at fixed temperature $k_{B}T=0.1$ eV for different values of frequency $\omega$ in the absence of strain.

We have studied the hole doping effects on the behavior optical absorption of $\text {MoS}_{2}$. For this purpose, the dependence of $Im(\epsilon (\omega ))$ on the negative chemical potential $\mu$ for different frequencies $\omega$ at fixed temperature $k_{B}T=0.1$ eV in the absence of magnetic field has been plotted in Fig. 10. A considerable peak in optical conductivity $\text {MoS}_{2}$ layer is clearly observed for $\omega =0.5$ eV. The chemical potential dependence for optical conductivity at $\omega =2.0$ eV indicates that $Im(\epsilon (\omega ))$ shows a monotonic increasing behavior with decrease of absolute value in the region $\mu <-1.0$. Upon more increase of $\mu$ above $-1.0$, optical conductivity for $\omega =2.0$ eV reaches a constant value according to Fig. 10.

**Figure 11:**
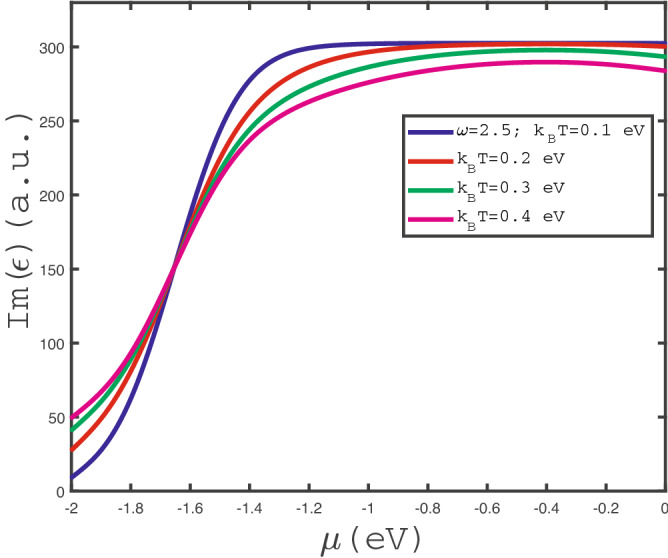
Optical absorption, $Im(\epsilon )$, of doped $\text {MoS}_{2}$ layer as a function of chemical potential $\mu$ at fixed frequency $\omega =2.5$ eV for different values of temperature, namely $k_{B}T=0.1~\text {eV},0.2~\text {eV},0.3~\text {eV},0.4~\text {eV}$, in the absence of strain.

In Fig. 11, we have presented the behavior of optical absorption of doped $\text {MoS}_{2}$ layer in terms of negative chemical $\mu$ in the absence of any strain parameter for different values of temperature. The photon has been fixed at $\omega =2.5$ eV. This plot indicates a monotonic increasing behavior for each value of temperature. It can be understood from the fact that the decrease of absolute value of chemical potential leads to reduce the hole concentration. Thus the scattering rate between holes decreases which increases the optical conductivity. Another feature is pronounced in this figure. For fixed chemical potential above $-1.6$ eV, $Im(\epsilon (\omega ))$ decreases with temperature according to Fig. 5. Higher temperature causes more scattering of holes which reduces the optical conductivity. However for chemical potentials below -1.6 eV, the increase of temperature leads to enhance the transition rate of electrons from valence band to conduction one. This fact implies optical conductivity increases with temperature in the chemical potential region below $-1.6$ eV.

**Figure 12:**
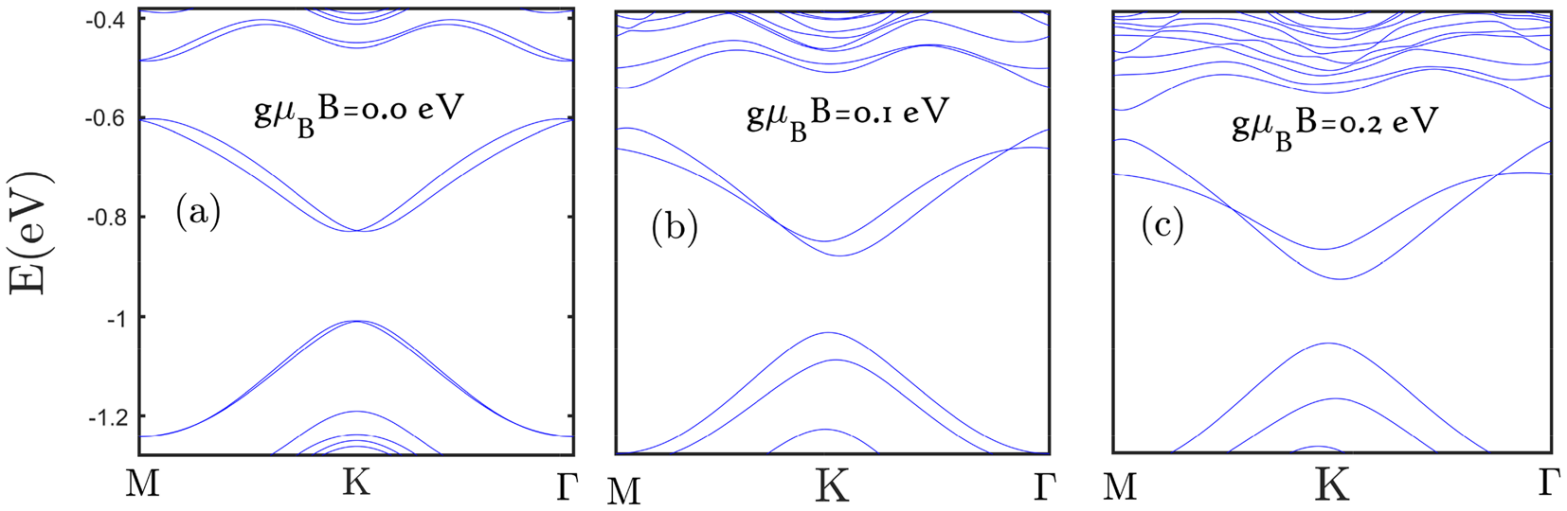
The electronic band structure of $\text {MoS}_{2}$ layer in the direction of M-K-$\Gamma$ into the first Brillouin zone in the absence of strain parameter with various magnetic fields, namely $g\mu _{B}B=0.0$ eV, 0.1 eV, 02 eV.

Finally we have plotted the electronic band structure of $\text {MoS}_{2}$ monolayer in the absence strain parameters for various external magnetic fields in Fig. 12. This figure implies the increase of applied magnetic field leads to decrease distance between energy levels as the structure tends to metallic property with magnetic field. Such metallic property with magnetic field has been approved in Fig. 2 where the Drude weight in optical conductivity increases with magnetic field.

## Data Availability

The datasets used and analysed during the current study available from the corresponding author on reasonable request.
